# Fluopack screening platform for unbiased cellular phenotype profiling

**DOI:** 10.1038/s41598-020-58861-3

**Published:** 2020-02-07

**Authors:** Zhao B. Kang, Ioannis Moutsatsos, Francesca Moretti, Phil Bergman, Xian Zhang, Beat Nyfeler, Christophe Antczak

**Affiliations:** 1 Novartis Institutes for BioMedical Research, Cambridge, MA USA; 2 Novartis Institutes for BioMedical Research, Basel, Switzerland

**Keywords:** High-throughput screening, Fluorescence imaging, Mechanism of action, Predictive markers, Phenotypic screening

## Abstract

Gene and compound functions are often interrogated by perturbation. However, we have limited methods to capture associated phenotypes in an unbiased and holistic manner. Here, we describe Fluopack screening as a novel platform enabling the profiling of subcellular phenotypes associated with perturbation. Our approach leverages imaging of a panel of fluorescent chemical probes to survey cellular processes in an unbiased and high throughput fashion. Segmentation-free, whole image analysis applied to Fluopack images identifies probes revealing distinct phenotypes upon perturbation, thereby informing on the function and mechanism of action of perturbagens. This chemical biology approach allows to interrogate phenotypes that tend to be overlooked by other methods, such as lipid trafficking and ion concentration inside the cell. Fluopack screening is a powerful approach to study orphan protein function, as exemplified by the characterization of TMEM41B as novel regulator of lipid mobilization.

## Introduction

Genome-wide association studies and genome-wide perturbation screens have linked many novel genes to diseases and cellular processes, but a large number of genes code for proteins of unknown or poorly characterized function. In order to attribute a function to such novel proteins, researchers can study their localization or identify interaction partners, but this approach is often limited by available tools such as antibodies. Alternatively, genetic or chemical perturbations can be exploited to modulate protein function before reading out the associated cellular phenotype. While transcriptional and proteomic profiling enable such unbiased analyses, more direct methodologies to rapidly and comprehensively characterize the cellular phenotypes of perturbations are still lacking.

**Figure 1:**
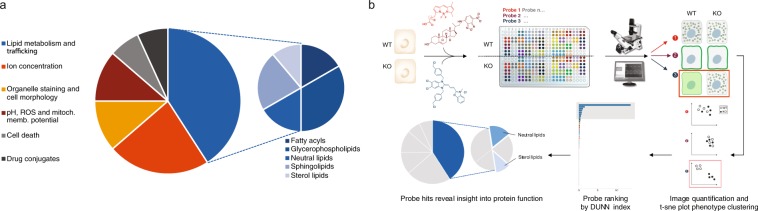
Overview of the Fluopack screening platform interrogating various cellular phenotypes to gain unbiased biological insight. (**a**) Distribution of high content imaging readout categories covered by the 44-probe Fluopack panel. (**b**) Overview of Fluopack screening workflow. In this example, Fluopack is used to compare cells with knocked-out expression of a protein of interest (KO) with wild-type cells (WT). Following addition of the probe panel with one probe per well, cells are imaged to reveal phenotypes. Those probes revealing a distinct phenotype between KO and WT cells are identified by image quantification and t-SNE clustering of phenotypes. A DUNN index is calculated to rank probes and prioritize images for visual inspection. The cellular phenotypes that top probes report on (e.g. neutral and sterol lipid trafficking) provides an insight into the biological function of the protein of interest. Drawings by Alan Abrams.

Here, we describe the development of a new platform for phenotype profiling relying on cellular high content imaging of a panel of fluorescent chemical probes, that we named Fluopack. This chemical biology approach utilizes 44 fluorescent chemical probes to read out the morphology of intracellular organelles, the endogenous concentration of different ions, cellular stress pathways, and the uptake and trafficking of different lipid classes (Fig. 1a, Suppl. Table S1). The Fluopack platform leverages high content imaging to identify subtle and complex phenotypes such as changes in the sub-cellular distribution or intensity of a given probe, in a high throughput fashion. In a typical profiling experiment aimed at characterizing the role of a given protein, parental cells are compared to cells with a gene knockout (KO). Both cell types are seeded onto the same 384-well plate, followed by addition of the probe panel with one probe per well. Cells are then imaged, and the entire process can easily be automated (Fig. 1b). The goal of Fluopack profiling in this context is to identify probes that reveal a distinct cellular phenotype associated with depletion of the protein of interest, in turn pointing to specific cellular processes modulated by the protein of interest.

**Figure 2:**
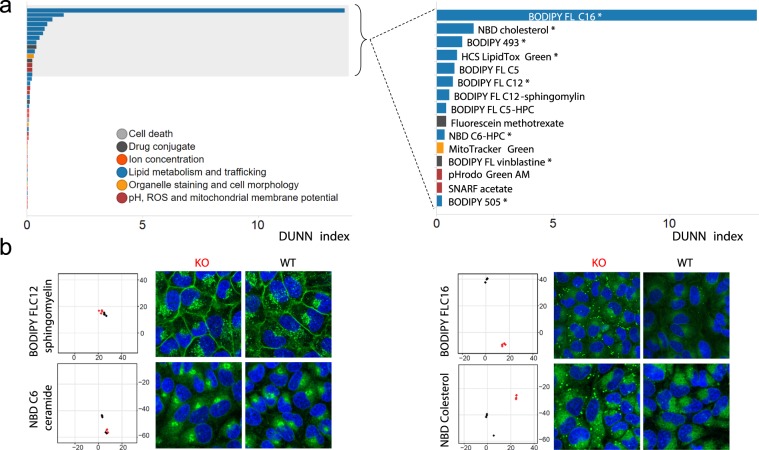
Outcome of the TMEM41B Fluopack screen. (**a**) DUNN index ranking of each probe tested in the TMEM41B Fluopack screen, color coded by probe category. The top 15 probes are shown on the right, with probes of interest as identified by visual inspection labeled with an asterisk (*). (**b**) Data for four representative probes, two non-hits (BODIPY FL C12 sphingomyelin, NBD C6 ceramide) and two hits (BODIPY FL C16, NBD cholesterol). For each probe, on the left is the t-SNE plot of the whole screen, zoomed in for the corresponding probe, and on the right are example images of TMEM41B KO and WT cells. BODIPY FL C16 and NBD cholesterol reveal specific accumulation of neutral lipids and cholesterol probes as puncta in TMEM41B KO cells.

As a proof of concept, we applied Fluopack screening to the characterization of TMEM41B, a largely uncharacterized transmembrane protein which scored as autophagy modulator in three independent pooled CRISPR screens^1–3^. We then visually reviewed all screening images to identify eight probes that reveal significant phenotypic changes between TMEM41B KO and WT cells (Table 1). Seven out of those eight selected probes report on lipids and reveal a striking puncta accumulation in TMEM41B depleted cells, especially for BODIPY 493, BODIPY FL C12 and NBD cholesterol (Fig. 2), as we previously described^2^. In order to capitalize on the holistic nature of the Fluopack approach, we sought to systematically evaluate and rank the potential phenotype modulation for all probes in our panel. Since multiple images are acquired for each probe and visual inspection is slow and only qualitative, we aimed to automate image quantification to identify probes of interest in a rapid and unbiased manner. However, a major limitation of traditional segmentation-based image quantification lies in the necessity of having prior knowledge of the phenotype to be quantified. In a phenotype profiling experiment comparing multiple probes, various phenotypes are typically observed that vary in intensity, patterns and subcellular localization. While such phenotypes can be identified upon visual inspection of images, the process is time-consuming, biased and not easily scalable. We overcame this limitation by applying a segmentation-free, whole image analysis algorithm to dissect and cluster those complex phenotypes, thus opening the door to automated, unbiased image quantification. The raw measurements of the CP-CHARM algorithm (Compound Hierarchy of Algorithms Representing Morphology) that we employed consist of 923 morphological image features that are subjected to feature reduction to remove features of low variance and high correlation and collinearity. The dimensionality of the 176 remaining features is reduced using t-Distributed Stochastic Neighbor Embedding (t-SNE)^4^. To identify probes that reveal distinct phenotypes induced by the perturbation, we review the 2D representation of the t-SNE feature embeddings (Suppl. Fig. 1). The t-SNE probe clusters can be further validated and ranked using a DUNN index^5^ internal cluster statistic to identify Fluopack probes that best separate experimental condition data clusters (Fig. 1b).

**Table 1:** Description of eight probes revealing phenotypic differences between TMEM41B KO cells, as identified by visual inspection.

SKU #	Truncated probe name	Category	Application
N1148	NBD cholesterol	Lipid metabolism and trafficking	Sterol lipids
D3922	BODIPY 493	Lipid metabolism and trafficking	Neutral lipids
H34475	HCS LipidTOX Green	Lipid metabolism and trafficking	Neutral lipids
D3921	BODIPY 505	Lipid metabolism and trafficking	Neutral lipids
D3821	BODIPY FL C16	Lipid metabolism and trafficking	Fatty acyls
D3822	BODIPY FL C12	Lipid metabolism and trafficking	Fatty acyls
N3786	NBD C6-HPC	Lipid metabolism and trafficking	Glycerophospholipids
V12390	BODIPY FL vinblastine	Drug conjugate	Tubulin staining

Applying our workflow to the comparison of TMEM41B WT and KO cells, we observed an enrichment of probes monitoring lipid metabolism and trafficking, with 10 probes in this category among the top scoring probes by DUNN index ranking (Fig. 2). All probes included in our panel that monitor fatty acyls (BODIPY FL C5, C12 and C16) ranked among the top 15 probes, as well as all probes staining endogenous neutral lipids (BODIPY 493, BODIPY 505 and HCS LipidTox Green). In addition, a probe for sterol lipids ranked number 2 (NBD cholesterol) (Fig. 2). This clear outcome from the CP-CHARM quantification is in line with our visual inspection of images, identifying a striking accumulation of neutral and sterol lipid probes in large puncta in TMEM41B-depleted cells. In contrast, other lipid probes such as sphingolipid and ceramide derivatives remained largely unchanged (Fig. 2). Altogether, CP-CHARM-based image quantification of this Fluopack screen confirms that TMEM41B KO specifically alters neutral and sterol lipid trafficking, consistent with the described role for TMEM41B in regulating lipid droplets, known to store these lipid classes inside the cells. Our previous study published elsewhere confirmed and validated those Fluopack observations^2^. As demonstrated in this study, the increase in lipid droplets in TMEM41B-depleted cells was substantiated by co-localization studies with the lipid droplet protein marker ADRP (Perilipin‐2) as well as by transmission electron microscopy^2^. Follow-up experiments with a red-fluorescent fatty acid analog revealed that TMEM41B is required for the efficient flux of fatty acid from lipid droplets to mitochondria^2^. Those observations demonstrate that Fluopack screening can identify phenotypes associated with protein function, for proteins that were previously poorly characterized.

While the CP-CHARM-based image quantification that we developed to quantify Fluopack screening images performed well, we sought to compare it with an unbiased segmentation-based approach. The purpose of this comparison was to assess whether a segmentation-based analysis could outperform the CP-CHARM approach, for example by identifying relevant probes that had been missed in our previous analysis. In the segmentation-based approach, approximated cell regions around stained nuclei are segmented and measured. The resulting 237 per object measurements are aggregated to a mean per image feature measurements (reduced to 97 features). When we compared the ranking of the probe panel resulting from CP-CHARM and segmentation-based analyses, we found that the CP-CHARM approach successfully highlighted the lipid trafficking category among the top probes, whereas no clear trend emerged from the segmentation-based analysis (Suppl. Fig. 2). Hence, the CP-CHARM analysis outcome best matches visual inspection. Furthermore, the top ranking probe by segmentation (heavy metal indicator Phen Green FL Diacetate) was found to constitute a false positive, since visual inspection of images revealed an absence of staining in both WT and KO TMEM41B cells (Suppl. Fig. 2). This result suggests that the segmentation-based approach was misled by low intensity fluorescent artifacts. Segmentation-based analysis also suffered from false negatives, as the visually confirmed probe BODIPY 505 was among the lowest ranked. In contrast, all eight top probes as selected by visual inspection (including BODIPY 505) were among the top 15 probes as ranked by CHARM analysis (Suppl. Fig. 2), demonstrating accurate ranking with this approach. Of note, three lipid probes among the top 15 were not selected by visual inspection, namely BODIPY FL C12-sphingomyelin, BODIPY FL C5 and BODIPY FL C5-HPC (Table 1, Fig. 2). Those probes likely constitute false positives of the CHARM analysis, but we also cannot rule out that our whole image-based approach picked up subtle differences missed by visual review. Based on this evaluation, we adopted the segmentation-free CP-CHARM approach to analyze Fluopack images, as it effectively identifies relevant phenotypes, and it does not require any manual customization nor fine-tuning for particular cell types or phenotypes. This latest point is of great value, since Fluopack is intended to be applied to diverse cellular systems and phenotypes.

**Figure 3:**
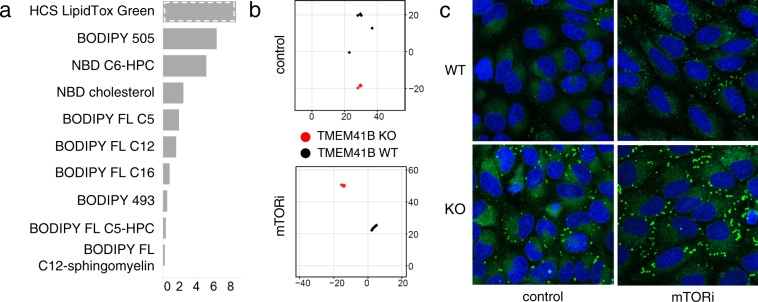
Modulation of observed phenotypes by mTOR inhibition. (**a**) Comparison of DUNN index ranking for the top 15 probes identified by CHARM analysis for baseline (DMSO control) and mTOR inhibition (mTORi, AZD8055), highlighting HCS LipidTox Green as the probe revealing the largest increase in DUNN index upon treatment. (**b**) t-SNE plots for HCS LipidTox Green probe highlighting increased separation of TMEM41B KO and WT phenotypes upon mTOR inhibitor treatment. (**c**) Images confirming the synergistic effect of TMEM41B KO and mTOR inhibition in inducing neutral lipid accumulation, as revealed by the HCS LipidTox Green probe.

Since both visual inspection of images and CP-CHARM analysis point to lipid trafficking as the main dysregulated cellular process upon TMEM41B deletion, we decided to focus on lipid probes for further characterization. For the 10 lipid probes identified among the top 15 probes as ranked by CP-CHARM analysis (Fig. 2), we compared the baseline phenotype in TMEM41B KO cells with the phenotype associated with the inhibition of mTOR, a key regulator of lipid metabolism^6^. For this purpose, we treated cells for 24 hours with the mTOR inhibitor AZD8055^7^ before adding the fluorescent probes. Images were submitted to CP-CHARM analysis, and DUNN indices were calculated for both conditions. Plotting the DUNN index fold change upon mTOR inhibition versus control treatment reveals an increase in DUNN index for several probes, most strikingly for the neutral lipid probe HCS LipidTox (Fig. 3a). This increase reflects an enhanced separation of the phenotype observed with TMEM41B KO cells compared to WT cells upon mTOR inhibitor treatment (Fig. 3b). Images confirm that the mTOR inhibitor AZD8055 accentuates puncta formation revealed by the HCS LipidTox probe (Fig. 3c), suggesting that TMEM41B depletion and mTOR inhibition act synergistically to amplify lipid accumulation in the observed puncta. We note that the most significant increases in DUNN index were observed for probes reporting on neutral and sterol lipids, confirming our previous observations that pointed out to a main role for TMEM41B in autophagy, related to lipid exchange from lipid droplets. Those results demonstrate that Fluopack screening can be applied to reveal phenotypes induced by low molecular weight perturbagens, in addition to gene knockout. Furthermore, we believe that such combination of genetic perturbation and small molecule modulation can be a powerful approach to identify synergistic or rescue effects in Fluopack screens, to confirm the involvement of specific cellular processes in observed phenotypes.

This proof of concept study demonstrates the value of Fluopack phenotype profiling for expanding our understanding of biology. While TMEM41B was identified as a novel regulator of autophagy in a CRISPR screen, its exact role was unknown. In a single profiling experiment, Fluopack provided a holistic view of phenotypes associated with TMEM41B KO (Fig. 2). As the probes report on specific cellular processes (Suppl. Table S1), the obtained phenotypic profile pointed directly to those cellular events that are affected by protein depletion (neutral lipid and cholesterol accumulation), and those that are not (accumulation of other lipid classes, organelle morphology). This result provided direct, actionable clues as to the function of TMEM41B. Orthogonal methods could then be applied to confirm and further characterize the role of TMEM41B in lipid mobilization from LDs, as described elsewhere^2^.

Altogether, our results suggest that Fluopack constitutes a complementary approach to other phenotype profiling platforms, by taking advantage of high content imaging of fluorescent chemical probes to systematically survey cellular phenotypes associated with a given perturbation. The unique value of our method lies in its holistic nature and ability to detect changes not captured by other profiling methods. By providing an unbiased “bird’s eye” view of phenotypes associated with perturbation, Fluopack can reveal the underlying biology when little or no information is previously available. In addition, we demonstrated that our approach can detect changes in lipid trafficking, which may not be associated with specific signatures in other high dimensional data sets such as transcriptomic analysis. Thus, Fluopack allows to capture phenotypes that could have easily been missed by other methods and constitutes a complementary approach to transcriptional and proteomic profiling. Other image-based phenotypic approaches are available. Simm *et al*. took advantage of features extracted from a 3-channel high content imaging assay to predict compound activity in unrelated assays^8^. Cell Painting inferred mode of action based on a set of six fluorescent stains revealing cellular morphology^9^. A potential advantage of Fluopack over those methods is its ability to uncover actionable phenotypes revealed by well-characterized probes reporting on defined cellular processes, that can be readily employed to further characterize function. In addition, Cell Painting and similar approaches require building a reference set of signatures with well-characterized perturbagens to connect morphological phenotypes to function, while Fluopack can be used in absence of such prior knowledge.

Even though our approach relying on fluorescent chemical probes could be prone to artifactual phenotypes driven by the fluorophore moiety, we address that concern by having designed built-in redundancy in our probe panel. Several versions of related probes coexist in the panel, giving higher confidence to Fluopack findings by minimizing the risk that observed phenotypes are caused by the fluorophore moiety of our probes. Following the same line of thought, we chose to minimize the potential risk for interference by exposing cells to only one probe at a time, instead of multiplexing different probes fluorescing at distinct wavelengths. This approach minimizes the risk that added probes interfere with cellular physiology, and eliminates the risk that multiple probes added to the same well would interfere with each other.

Applications of Fluopack expand beyond the current use case described here, due to its versatility. We have shown that our approach is not limited to genetic perturbation and can instead be applied to survey cellular phenotypes associated with a chemical perturbation, opening the door to characterizing the mode of action of low molecular weight compounds of interest. Furthermore, the type of biological processes that can be interrogated by the platform is only limited by the availability of relevant fluorescent chemical probes. As new probes revealing different aspects of biology become available, they can easily be integrated to the platform. As such, we have recently expanded our probe panel from 44 to 170 probes. Example areas of biology that can be studied with additional commercially available probes are protein detection, protein uptake and trafficking, glycoconjugate detection, and carbohydrate uptake. Importantly, relying on exogenously added fluorescent chemical probes ensures that the platform is readily deployable in any cell model including primary cells, since no cell engineering or antibody staining is required for readout. Finally, Fluopack screening with cell-permeable probes can deliver live kinetic data that further expands our understanding of the biology affected by a given perturbation. In that mode, researchers need not guess the relevant time point and instead can track changes in phenotype over time, in the same cell. This feature overcomes a limitation of transcriptional and proteomic profiling, that require replicate experiments for different time points.

We anticipate that the most valuable feature of Fluopack will be its ability to reveal subcellular phenotypes easily missed by other methods, such as changes in lipid trafficking inside the cell, or variations in the concentration of ions in specific organelles. Growing evidence suggests that dysregulation of lipid metabolism occurs in diseases such as Alzheimer’s^10^ and Parkinson’s disease^11^. This type of alteration had been mostly overlooked so far, for lack of convenient tools to enable their study. We believe that Fluopack could fill that gap, with a broadly applicable, complimentary and scalable approach to characterize proteins and compounds of interest. Finally, we foresee that new developments in image analysis methods such as deep learning^12^, coupled with to Fluopack cellular profiling could considerably augment our characterization of disease phenotypes.

## Methods

### Fluopack screening

H4 Cas9 cells stably expressing TMEM41B or non-targeting sgRNAs, were seeded at 3.5 × 10^3^ cells per well onto assay plates (Perkin Elmer CellCarrier 384-well microplates) in 40 µL growth media. A panel of fluorescent chemical probes was obtained from Thermo Fisher Scientific (Suppl. Table 1) and plated into source plates (Greiner bio-one 384-well microplates) in DMSO. Since the relevant concentration range varies for each probe, we curated the panel of probes and annotated the list with the optimal range of concentration for each probe, as per the manufacturer recommendation. Probes were then pre-plated accordingly at a source concentration of 200X, leading to a final concentration X within the optimal range for each probe. Each probe was plated in quadruplicate, and transferred from source plates into assay plates by acoustic dispensing of 200 nL. Hoechst nuclear stain was added to the assay plates in 2 µL DMEM using Multidrop Combi (Thermo Fisher Scientific, Model# 836). After 2 h incubation at 37 °C, cells were imaged live at 60X objective magnification on a Cell Voyager 7000S automated confocal microscope, yielding 3 fields per well. Since two technical replicates were imaged for each condition (WT vs. KO), a total of 12 images were acquired for each probe. A screen was performed in parallel where probes were added to fixed cells instead of live cells, to reveal any phenotype for those probes that are not cell permeable (data not shown). To study the effect of mTOR inhibition, the low molecular compound AZD8055 was added to cells for 24 hours prior to probe addition, at a 500 nM final concentration in 0.4% DMSO (v/v). Wells treated with mTOR inhbitor were compared to wells treated with 0.4% DMSO (v/v) as a control.

### Image analysis and quantification

Images were analyzed using CellProfiler v2.2.2 on a Linux high performance compute cluster using the Jenkins-LSCI framework as previously described^13^. For segmentation-free image analysis, image features were computed on the whole image using the CellProfiler CHARM modules^14^. As of this writing, the CHARM modules are not included in the official release of CellProfiler, but the modules and the image feature extraction pipeline ([TrainTestMode]CHARM-like) can be obtained from the CPCharm git repository^15^. The CHARM pipeline extracts a total of 923 morphological image features that can be used for image classification and clustering. For segmentation-based image analysis we first identified nuclei in the DNA channel, and then approximated the cell regions using the Distance-N method of the CellProfiler IdentifySecondaryObjects module. We generated measurements of Granularity, Object Neighbors, Object Intensity Distribution, Object Intensity and Texture within these cell regions from the probe channel. The per-object measurements are aggregated to generate 237 per image mean measurement values. These per-image mean values are then processed similarly to CHARM per image measurements. The features were analyzed using the NIBR Multi-parametric Data Analysis (MPDA) system that performs analyses for feature correlation, collinearity and variance. MPDA selects features that are deemed most informative for discriminating positive and negative control images, and the same type of analysis could be performed using the open source alternative KNIME Open Analytics Platform^16^. After feature reduction, a total of 176 image features were retained. The reduced feature set was annotated with experimental metadata (such as cell-line, treatment, probe names, and probe SKUs) and used for t-distributed Stochastic Neighbor Embedding (t-SNE) analysis and visualization^4^. Graphical visualization of the 2D features generated from images from the same probe using the t-SNE embeddings was used to identify probes that yield distinct phenotypes between the TMEM41B KO and WT cells. The Dunn Index^5^ (calculated from the t-SNE embeddings) was also used to quantify how well image clusters were separated. The t-SNE analysis was performed in R version 3.5.2 using the “Displayr/flipDimensionReduction” package^17^.

## Supplementary information

Supplementary Information
