# Supplementary material for: Fluopack screening platform for unbiased cellular phenotype profiling
Source: Sci Rep. 2020 Feb 7;10:2097. doi: 10.1038/s41598-020-58861-3 (PMC7005823; doi:10.1038/s41598-020-58861-3)
Supplement: Supplementary file 1 — Supplementary Information [file 41598_2020_58861_MOESM1_ESM.docx]

**Fluopack screening platform for unbiased cellular phenotype profiling**

Zhao B. Kang^1^, Ioannis Moutsatsos^1^, Francesca Moretti^2^, Phil Bergman^1^, Xian Zhang^2^, Beat Nyfeler^2^, Christophe Antczak^1^*

^1^Novartis Institutes for BioMedical Research, Cambridge, MA, USA

^2^Novartis Institutes for BioMedical Research, Basel, Switzerland

Supplementary information

**Supplementary Table S1.** List of probes included in the Fluopack panel sorted by category, along with description and tested final concentration.

| **SKU #** | **Truncated probe name** | **Category** | **Application** | **final concentration (M)** |
| --- | --- | --- | --- | --- |
| C10423 | CellEvent Caspase Green | Cell death | Caspase activated DNA staining | 2.00E-06 |
| I10291 | Image-iT DEAD Green | Cell death | Viability stain impermeant to healthy cells | 1.00E-07 |
| S7020 | SYTOX Green | Cell death | Cell impermeant nuclear stain, dead cell staining | 1.00E-07 |
| M1198MP | fluorescein methotrexate | Drug conjugate | Methotrexate tracking, DHFR expression | 2.00E-06 |
| V12390 | BODIPY FL vinblastine | Drug conjugate | Vinblastine conjugate, tubulin staining | 2.00E-06 |
| V34850 | BODIPY FL vancomycin | Drug conjugate | Vancomycin conjugate, gram positive bacteria staining | 5.80E-07 |
| F23915 | fluo-3 AM | Ion concentration | Calcium probe | 5.00E-06 |
| F23917 | fluo-4 AM | Ion concentration | Calcium probe | 5.00E-06 |
| F23981 | fluo-4FF AM | Ion concentration | Calcium probe | 5.00E-06 |
| F24195 | FluoZin-3 AM | Ion concentration | Zinc probe | 5.00E-06 |
| M14206 | mag-fluo-4 AM | Ion concentration | Magnesium/calcium probe | 5.00E-06 |
| M3735 | Magnesium Green AM | Ion concentration | Magnesium probe | 5.00E-06 |
| N7991 | Newport Green DCF diacetate | Ion concentration | Zinc probe | 5.00E-06 |
| O6807 | Oregon Green BAPTA-1 | Ion concentration | Calcium probe | 5.00E-06 |
| P6763 | Phen Green FL diacetate | Ion concentration | Ions, broad range | 2.00E-06 |
| S6901 | Sodium Green | Ion concentration | Sodium probe | 1.00E-05 |
| B13950 | BODIPY FL C5-ganglioside GM1 | Lipid metabolism and trafficking | Sphingolipids | 4.75E-07 |
| C3927MP | cholesteryl BODIPY FL C12 | Lipid metabolism and trafficking | Sterol lipids | 5.00E-06 |
| D3522 | BODIPY FL C5-sphingomyelin | Lipid metabolism and trafficking | Sphingolipids | 5.00E-06 |
| D3803 | BODIPY FL C5-HPC | Lipid metabolism and trafficking | Glycerophospholipids | 5.00E-06 |
| D3821 | BODIPY FL C16 | Lipid metabolism and trafficking | Fatty acyls | 1.00E-06 |
| D3822 | BODIPY FL C12 | Lipid metabolism and trafficking | Fatty acyls | 1.00E-06 |
| D3834 | BODIPY FL C5 | Lipid metabolism and trafficking | Fatty acyls | 1.00E-06 |
| D3921 | BODIPY 505 | Lipid metabolism and trafficking | Neutral lipids | 5.00E-06 |
| D3922 | BODIPY 493 | Lipid metabolism and trafficking | Neutral lipids | 5.00E-06 |
| D7711 | BODIPY FL C12-sphingomyelin | Lipid metabolism and trafficking | Sphingolipids | 5.00E-06 |
| F362 | fluorescein DHPE | Lipid metabolism and trafficking | Glycerophospholipids | 5.00E-06 |
| H34475 | HCS LipidTOX Green | Lipid metabolism and trafficking | Neutral lipids | 5.00E-06 |
| N1148 | NBD cholesterol | Lipid metabolism and trafficking | Sterol lipids | 5.00E-06 |
| N1154 | NBD C6-ceramide | Lipid metabolism and trafficking | Sphingolipids | 5.00E-06 |
| N360 | NBD-PE | Lipid metabolism and trafficking | Glycerophospholipids | 5.00E-06 |
| N3786 | NBD C6-HPC | Lipid metabolism and trafficking | Glycerophospholipids | 5.00E-06 |
| N3787 | NBD C12-HPC | Lipid metabolism and trafficking | Glycerophospholipids | 5.00E-06 |
| O12650 | Oregon Green DHPE | Lipid metabolism and trafficking | Glycerophospholipids | 5.00E-06 |
| E12353 | ER-Tracker Blue-White | Organelle staining and cell morphology | ER staining | 1.00E-06 |
| E34251 | ER-Tracker Green | Organelle staining and cell morphology | ER staining | 1.00E-06 |
| L7526 | LysoTracker Green | Organelle staining and cell morphology | Lysosome staining | 2.00E-08 |
| M7514 | MitoTracker Green | Organelle staining and cell morphology | Mitochondria staining | 2.00E-08 |
| T34075 | TubulinTrackerGreen | Organelle staining and cell morphology | Tubulin staining | 2.50E-07 |
| A1372 | acridine orange | pH, ROS and mitochondrial membrane potential | Mitochondrial membrane potential probe | 2.12E-06 |
| C10444 | CellROX Green | pH, ROS and mitochondrial membrane potential | ROS activated DNA staining | 2.50E-06 |
| P35373 | pHrodo Green AM | pH, ROS and mitochondrial membrane potential | pH indicator | 5.00E-06 |
| S23923 | SNARF acetate | pH, ROS and mitochondrial membrane potential | pH indicator | 1.00E-05 |
| S36002 | Singlet Oxygen Sensor Green | pH, ROS and mitochondrial membrane potential | ROS | 5.00E-06 |

**Suppl. Figure 1.** Image and data analysis workflow.

**
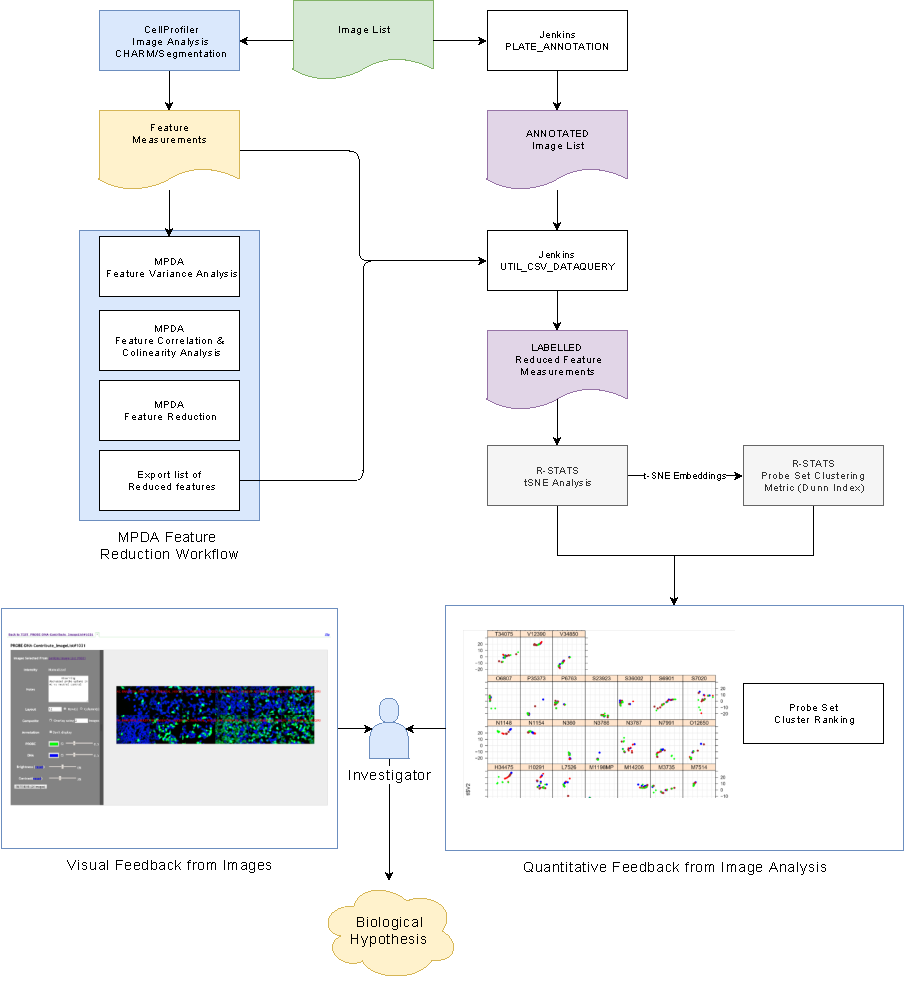
**

**Suppl. Figure 2**. DUNN index comparison of probe ranking for CP-CHARM- and segmentation-based analysis of the TMEM41B Fluopack screen.

**
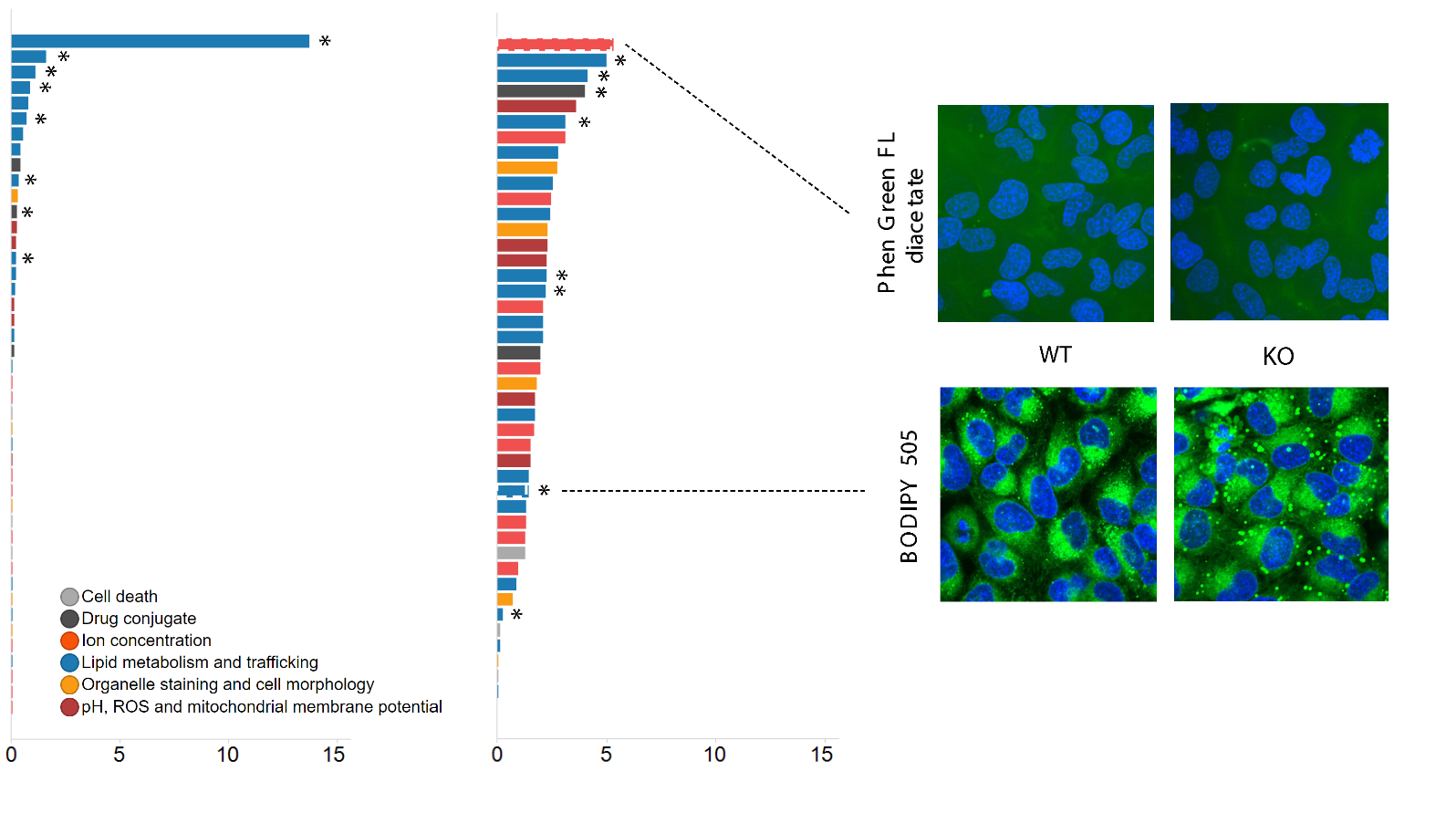
**
